# Influence of the COVID‐19 pandemic on amphibian road mortality

**DOI:** 10.1111/csp2.535

**Published:** 2021-09-29

**Authors:** Gregory LeClair, Matthew W. H. Chatfield, Zachary Wood, Jeffrey Parmelee, Cheryl A. Frederick

**Affiliations:** ^1^ School of Biology and Ecology University of Maine System Orono Maine USA; ^2^ Department of Biology University of New England Biddeford Maine USA; ^3^ Center for Wildlife Studies North Yarmouth Maine USA

**Keywords:** amphibians, community science, COVID‐19, mortality, road, traffic, transportation

## Abstract

The COVID‐19 pandemic and its related human activity shutdowns provide unique opportunities for biodiversity monitoring through what has been termed the “anthropause” or the “great human confinement experiment.” The pandemic caused immense disruption to human activity in the northeastern United States in the spring of 2020, with notable reductions in traffic levels. These shutdowns coincided with the seasonal migration of adult amphibians, which are typically subject to intense vehicle‐impact mortality. Using data collected as part of an annual community science monitoring program in Maine from 2018 to 2021, we examined how amphibian mortality probabilities responded to reductions in traffic during the pandemic. While we detected a 50% decline for all amphibians, this was driven entirely by reductions in frog mortality. Wildlife collision data from the Maine Department of Transportation on other wildlife species support our finding of drastic declines in wildlife road mortality in spring 2020 when compared with immediately previous and subsequent years. Additionally, we find that frogs suffer significantly higher road mortality than salamanders, particularly when conditions are warmer and wetter.

## 1 INTRODUCTION

The COVID‐19 pandemic caused a rare, sudden shift in global human activity due to restrictions emplaced by governments worldwide to contain the virus, especially during the initial months of the outbreak. These restrictions have provided an unlikely opportunity to observe ecological responses to an ultimately less active human population (Bates, Primack, Moraga, & Duarte, 2021). A growing body of evidence in the scientific literature suggests the pandemic is having mixed effects on wildlife populations (Manenti et al., 2020; Rondeau, Perry, & Grimard, 2020). Negative impacts stemming from COVID‐19 shutdowns include increases in some air pollutants (Higham, Ramirez, Green, & Morse, 2020), increases in traffic noise levels due to fewer but faster moving vehicles (Stokstad, 2020), increased illegal activity due to a reduction in law enforcement (Manenti et al., 2020), and reduction or halting of conservation programs (Magalhães et al., 2020). However, evidence of immediate net positive effects on wildlife are numerous and include detection of rare species in urban areas suggesting possible range expansion (Silva‐Rodríguez, Gálvez, Swan, Cusack, & Moreira‐Arce, 2021; Simon, 2020; Stokstad, 2020), exploitation of newly available resources (Derryberry, Phillips, Derryberry, Blum, & Luther, 2020), and improved recruitment and survivorship (Manenti et al., 2020; Shilling et al., 2021). Among changes in human behavior resulting from the COVID‐19 pandemic was a major reduction in travel (Pepe et al., 2020). This reduction in human movement, which has been termed the “anthropause” (Rutz et al., 2020) or “great human confinement experiment” (Bates et al., 2021) may have directly influenced wildlife populations in positive ways (Manenti et al., 2020; Shilling et al., 2021; Stokstad, 2020).

Of the benefits from reductions in traffic, perhaps no group stood to gain more from reduced road mortality than amphibians (e.g., Fahrig & Rytwinski, 2009; Glista, DeVault & DeWoody, 2008). It is well documented that roads have had profound consequences for amphibians (reviewed in Beebee, 2013), with problems exacerbated in areas with higher road density and traffic (Fahrig, Pedlar, Pope, Taylor, & Wegner, 1995; Gibbs & Shriver, 2005; Mazerolle, 2004) due to the propensity for many species to move onto roadways during migrations and specific weather events (e.g., Bingham, Marcum, & Morgan, 2018), changes in habitat (Cosentino et al., 2014), and sensitivity to road‐related pollutants (Karraker, Gibbs, & Vonesh, 2008). Given how heavy mortality from these forces may be, localized extirpations are possible (e.g., Gibbs & Shriver, 2005; Karraker et al., 2008). Roads passing through rural areas with little development can have substantial impacts on amphibians (Sillero, 2012), even unpaved roads with relatively low traffic (deMaynadier & Hunter Jr, 2000). This translates to the regional scale as well, as documented using data generated by a community science project (Cosentino et al., 2014).

Community science projects (until recently referred to as “citizen science”) are a growing component of many large‐scale ecological research programs. Volunteers of various backgrounds participate in scientist‐led projects, thereby increasing overall data collection effort. By expanding spatial and temporal data collection opportunities, they may provide insights into ecological trends that would otherwise be difficult to obtain (Walker & Taylor, 2017). Due to its scale, community science has become a viable option for assessing COVID‐19 impacts on nature (Bates et al., 2021; Vardi, Berger‐Tal, & Roll, 2021) and could be useful in untangling complicated relationships attributed to the pandemic.

The Maine Big Night: Amphibian Migration Monitoring (hereafter “MBN”) is a community science project that collects data on migrating amphibians crossing roads at sites throughout the state of Maine in the northeastern United States. The program enables detection of sites with significant road mortality, reduction of mortality events through intervention, and monitoring of populations over time. We capitalized on data collected as part of this ongoing effort to test our hypothesis that there was a positive impact of the COVID‐19 pandemic on amphibians via reduced road mortality. We further examined this relationship by comparing mortality probabilities between salamanders and frogs to determine if pandemic‐related shutdowns affected these groups equally or whether order‐specific differences existed. Since the project has a relatively short‐term dataset, we compiled broader support for the benefits provided by shutdowns on local wildlife by including roadkill data collected by the Maine Department of Transportation (MDOT) on other Maine wildlife species.

## 2 METHODS

### 2.1 Community science project

We used data from the MBN community science project for spring 2018–2021. Volunteers were trained, either online or in‐person, in amphibian identification, data collection methods, and safety. Following training, participants were required to achieve a passing score (>80%) on an online exam to be considered certified and given a choice of survey sites to adopt. We preselected survey sites based on previous observations of amphibian movements, migratory amphibian reports to the iNaturalist web‐based platform, or a geographic information system model that identified potential migratory locations based on roads within ~300 m from identified significant vernal pools (see Maine Department of Environmental Protection [MDEP] website for definitions; maine.gov/dep/land/nrpa/index.html) (Maine Department of Inland Fisheries and Wildlife, 2020). Sites were 300 m stretches of road and centered on expected highest amphibian migratory traffic locations. Site length and selection was based on typical migratory movement distances of target species such as wood frogs (*Lithobates sylvaticus*) (Baldwin, Calhoun, & deMaynadier, 2006) and blue‐spotted salamanders (*Ambystoma laterale*) (Hoffmann, Hunter Jr, Calhoun, & Bogart, 2018). As of the end of the 2021 season, 432 sites were available for adoption. Nearly all amphibian migration activity occurs in early to mid‐spring in Maine, which we have defined as the survey window from March 15 to May 15. Despite the project name referring to a singular night, migrations typically occur over multiple nights and thus volunteers were encouraged to survey as many suitable nights as possible. Specific timing was up to the volunteers; however; surveying on nights with forecasts for precipitation and temperatures at or above 7.2°C was strongly advised. Certified volunteers occasionally participated with other certified volunteers and were often accompanied by uncertified volunteers (e.g., friends and family) who assisted in locating amphibians. During surveys, only one certified volunteer recorded data at a time to prevent overlap and double counts in the dataset.

Our protocol for data collection was as follows: volunteers would immediately begin visual encounter surveys for amphibians by walking the entirety of the 300 m transect and tallying the number of alive, injured, and dead individuals found, identifying each to species level when possible. Since part of the project's goals are to alleviate mortality pressure, live individuals were moved out of the roadway and onto the road shoulder in their direction of travel. All dead individuals were removed from the roadway to avoid double counting. Volunteers were asked to survey for at least 1 hr at each site adopted during the survey season. For the 2018 season, end‐times were not a required data field so we used photograph timestamps or a conservative 30 min after start time for missing end‐time datapoints.

We evaluated and ranked (high to low) every submission according to set criteria for quality (e.g., adherence to collection protocols, probability/validity of observations, and completeness of data entry) to minimize interparticipant variation and data recording errors. High quality data were accepted, medium quality data were further investigated to resolve minor issues, and all nonresolvable and low‐quality data were removed along with observations when no amphibian sightings occurred. We ultimately replaced the subjective metrics reported for weather designations with local weather station data from the National Oceanic and Atmospheric Administration's website (NOAA, 2021) for maximum daily temperature and daily precipitation at the county level, averaging across stations whenever there was more than one station in a single county or using stations in the nearest neighbor county when stations in the target county were offline.

### 2.2 Amphibian individual mortality probabilities

We examined amphibian individual mortality probabilities—*the probability of a single individual suffering mortality during a road crossing*. Individual mortality probability—by virtue of being applied at the individual level—is independent of the number of individuals surveyed, which could vary due to surveying effort or population fluctuations. We analyzed individual mortality probability using generalized linear mixed models (GLMM) with a binomial distribution in R (Bates, Mächler, Bolker, & Walker, 2015; R Core Team, 2019), with individual mortality values (0 = survived; 1 = died) as our response variable. We included fixed effects of year (categorical), maximum daily temperature at the county level (linear), and daily precipitation at the county level (linear), and random effects for sample sites and recorders to remove bias from differences in sample sizes and recorder ability in our models. We fit the following model separately for frogs and salamanders, and again for all amphibians together:

(1) $$M=\beta_{Y}+\beta_{C}C+\beta_{P}P+\sigma_{\text{Site}}^{2}+\sigma_{\text{Recorder}}^{2}$$

where *M* is the individual mortality probability; *β*_*Y*_ is a year‐specific (*Y*) mortality probability (fixed effect); *β*_*C*_ *C* is a fixed effect of maximum temperature (*C*); *β*_*P*_ *P* is a fixed effect of daily precipitation (*P*); and *σ* ^2^_Site_ and *σ* ^2^_Recorder_ are the random effects for site and recorder identities, respectively. *C* and *P* were scaled (set to mean = 0 and *SD* = 1) for this analysis. We used Type II likelihood ratio tests to test for significant effects of year, temperature, and precipitation on individual mortality probability. We used Tukey post hoc tests (Hothorn, Bretz, & Westfall, 2008) to examine differences between individual mortality probability estimates for specific years.

### 2.3 Deer–vehicle collisions

The MDOT monitors vehicle‐animal collisions for a variety of wildlife species, including deer, bear, moose, turkeys, and other species. We obtained data for the months of March and April for the years 2018–2021 through the MDOT public collision query tool (MDOT, 2021) to place our amphibian data in a broader context. Since other species had only a few data points per year, white‐tailed deer (*Odocoileus virginianus*) were compared separately from all other species due to the abundance of data for deer collisions.

### 2.4 Traffic

To examine the influence of traffic levels on amphibian mortality probabilities, we gathered publicly available data on traffic from Maine highway tollbooths (Maine Turnpike Authority, 2021) as well as localized state and rural routes from MDOT counting stations (MDOT Traffic Data, 2021). We examined interannual differences in relative traffic volume (volume divided by the 2018–2021 average to account for some roads being busier than others) using general linear models, fit separately for March and April data:

(2) $$\ln\left(\frac{V_{R_{Y}}}{\bar{V_{R}}}\right)=\beta_{Y}$$

where *V*_*RY*_ = traffic volume for a given road (*R*) for a given year (*Y*), *V*_*R*_‐bar = average (2018–2021) traffic volume for a given road (*R*), and *β*_*Y*_ = a year‐specific traffic coefficient. We used Type II likelihood ratio tests to test for significant effects of year on relative traffic volume. We used Tukey post hoc tests (Hothorn et al., 2008) to examine differences between relative traffic volume estimates for specific years.

### 2.5 Relationship between traffic and mortality

We examined relationships between March and April average traffic volume, amphibian individual mortality probabilities, and deer–vehicle collisions using a correlation matrix. Our traffic volume input was yearly average March and April relative traffic volume (*β*_*Y*_ from Equation (2), averaged across March and April models). Our amphibian individual mortality probability inputs were estimated yearly individual mortality probabilities for frogs and salamanders (logit(*β*_*Y*_) from Equation (1), calculated separately for frogs and salamanders). Our deer–vehicle collision input was the ln‐transformed number of collisions in March and April of each year. We calculated the correlation coefficient (*r*) for each pairwise combination of inputs above. We did not conduct significance tests on these correlations, as each correlation only had four data points (one per year).

## 3 RESULTS

### 3.1 Community science project

Since 2018, MBN has hosted 426 certified volunteers, with roughly one‐third (*n* = 149) of the documented volunteers (i.e., identified as the primary data submitter) contributing data throughout the study period as the primary data submitter, along with an unknown number of other volunteers (i.e., who were not submitters). Of the 432 sites available, 199 were surveyed by volunteers and had at least one amphibian detected during the survey period. Sites that produced amphibians were surveyed between 1 and 13 times throughout the study period. Total reported survey time (survey hours × participating certified volunteers) for all 3 years was 867.6 hr (Table 1), ranging from 10 to 180 min per survey (mean = 43.0).

**TABLE 1:** Volunteer involvement and effort across years

Year	Number	Number certified	Total
	Sites surveyed	Volunteers^a^	Volunteer hours
2018	4	NA^b^	17.5
2019	13	23	25.5
2020	62	87	87.5
2021	137	316	737.1

^a^ Not all certified volunteers participated or were officially recorded as a participant (i.e., participated with another volunteer who submitted data on behalf of both).

^b^ 2018 total certification numbers were not documented.

### 3.2 Amphibian individual mortality probabilities

**FIGURE 1:**
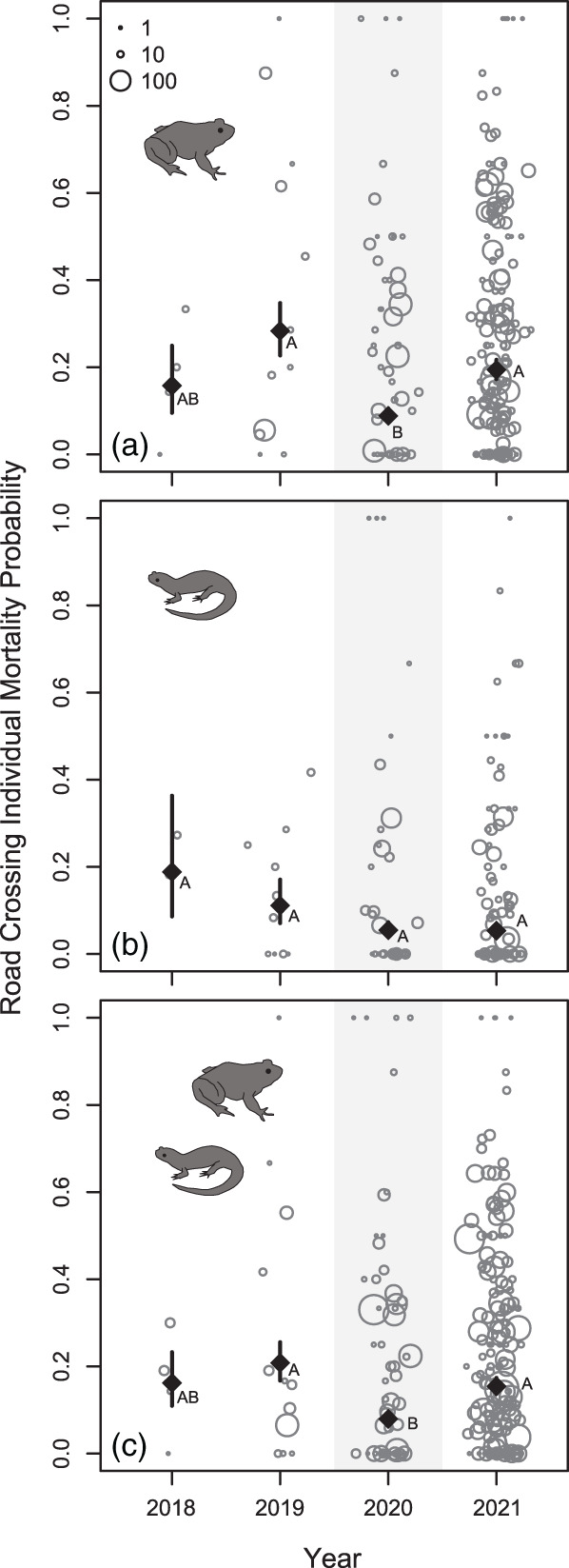
Amphibian individual mortality probability site‐specific averages across years separated by (a) frogs, (b) salamanders, and (c) frogs and salamanders combined. Each point represents the average individual mortality probability for a single site for a given year. Larger points indicate site + year combinations with a larger amphibian sample size. Dark points and bars show generalized linear mixed model predictions and *SE*s, respectively. Letters indicate classification of years based on Tukey post hoc tests. Mortality probabilities data also used in unpublished submitted manuscript Bates et al. “Global COVID‐19 lockdown highlights humans as threats and custodians of the environment”

**FIGURE 2:**
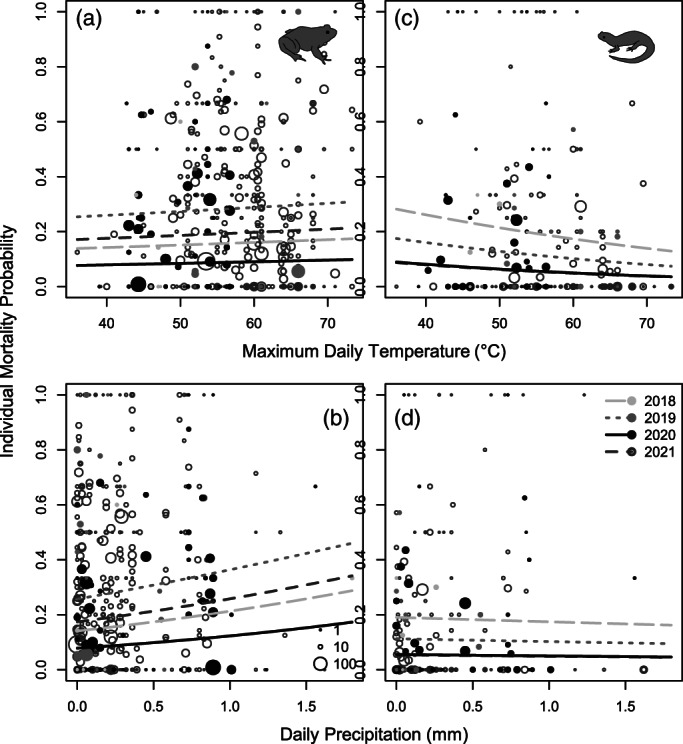
Responses of amphibian individual mortality probabilities to weather variables. (a,b) Correspond to frog mortality probabilities compared with temperature and precipitation, respectively, and (c,d) correspond to salamander mortality probability responses to temperature and precipitation, respectively. Each point represents the average individual mortality probability for a single site on a single night. Larger points indicate site + night combinations with a larger amphibian sample size. Lines indicate generalized linear mixed models (GLMM) year‐specific predictions

Our GLMM showed significant differences across years in individual mortality probabilities, predominantly driven by frog mortality. Over the course of the study, 7,749 amphibians were recorded representing 16 species (Supporting Information). According to likelihood ratio tests for model variables, frog, but not salamander road crossing individual mortality probabilities showed significant interyear variation (Figure 1; Table 2), driven by a roughly 50% decrease in mortality probabilities from 2020 compared to the other three survey years (Table 3). Interestingly, frogs also exhibited significantly higher individual mortality probabilities than salamanders across years (Figure 1; Table 3). Increasing precipitation corresponded to higher mortality probabilities in frogs, but not salamanders (Figure 2; Table 2).

**TABLE 2:** Type II likelihood ratio tests for amphibian road crossing individual mortality probability model variables

Model	Variable	*χ* ^2^	*df*	*p*
All	Year	36.23	3	**<.001**
	Maximum temperature	0.22	1	.64
	Daily precipitation	4.83	1	**.028**
Frogs	Year	41.04	3	**<.001**
	Maximum temperature	0.77	1	.38
	Daily precipitation	7.47	1	**.006**
Salamanders	Year	4.89	3	.18
	Maximum temperature	2.12	1	.15
	Daily precipitation	0.08	1	.77

*Note*: All are considered significant as out chosen alpha level is 0.05.

**TABLE 3:** Estimated mean amphibian road crossing individual mortality probabilities from generalized linear mixed models, with lower and upper *SE* bounds. Mortality probability data for years 2018–2020 also used in unpublished accepted manuscript Bates et al. “Global COVID‐19 lockdown highlights humans as threats and custodians of the environment”

Taxa	Year	Estimate	Lower	Upper
All	2018	16.2	11.0	23.3
	2019	20.8	16.7	25.6
	2020	7.9	6.7	9.4
	2021	15.4	13.7	17.4
Frogs	2018	15.8	9.5	24.9
	2019	28.3	22.7	34.7
	2020	8.8	7.4	10.5
	2021	19.4	17.3	21.7
Salamanders	2018	18.8	8.6	36.3
	2019	11.1	7.1	17.1
	2020	5.5	4.1	7.3
	2021	5.3	4.2	6.7

### 3.3 Deer–vehicle collisions

**FIGURE 3:**
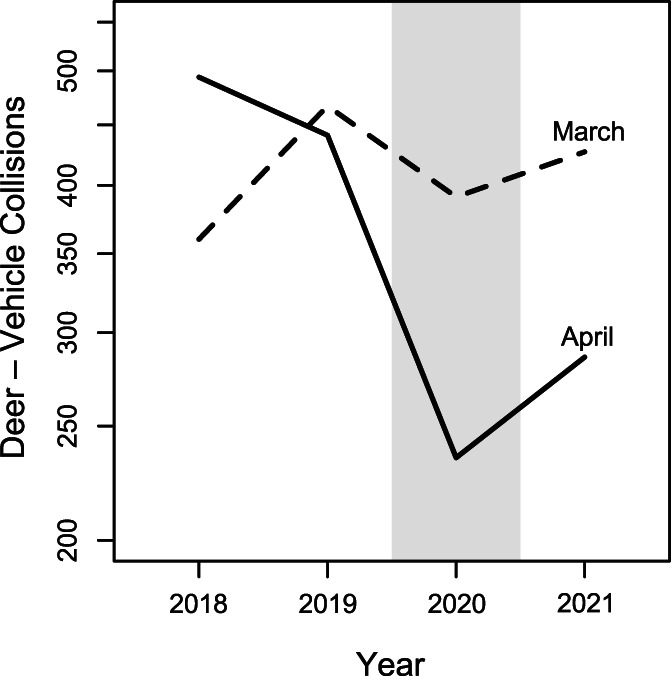
Statewide deer–vehicle collision reported by the Maine DOT in March and April for years 2018–2021

Vehicle collisions with white‐tailed deer dropped by 53% from April 2019 to 2020, with a smaller drop in March (Figure 3). Deer–vehicle collisions then increased from 2020 to 2021 (Figure 3). Other wildlife collision data were consistent with this trend and generally followed this pattern of sharp declines in road‐related mortality in spring 2020 produced by our community science data set, even in low‐sample species such as wild turkey (*Meleagris gallopavo*) which showed a 58% decline and moose (*Alces alces*) which showed a 50% decline (Supporting Information).

### 3.4 Traffic

**FIGURE 4:**
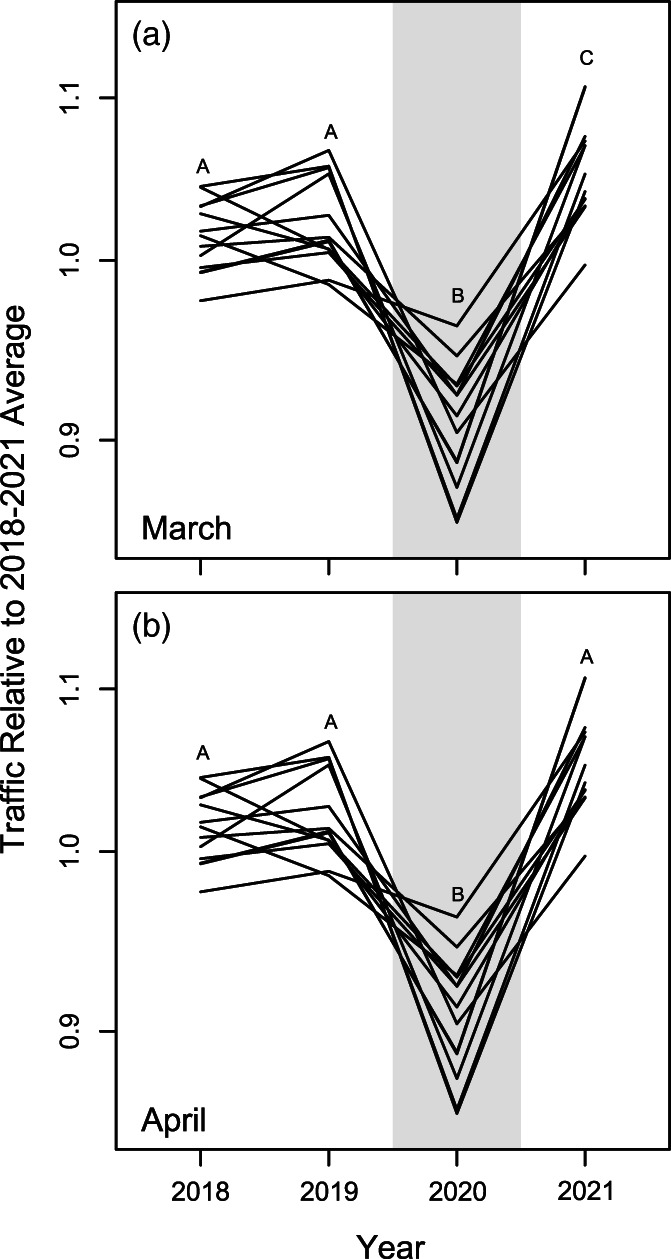
Relative traffic volume for several rural roads in Maine for (a) March and (b) April from 2018 to 2021. Each line indicates a single road. Letters indicate classification of years based on Tukey post hoc tests. For list of roads, see supplemental materials

Relative traffic volume on Maine roads varied significantly from year to year in both March (likelihood ratio test: *χ* ^2^ = 206.09; *df* = 3; *p* < .001; Figure 4) and April (*χ* ^2^ = 240.82; *df* = 3; *p* < .001; Figure 4). This variation was largely driven by a traffic decline of 15–20% from 2019 to 2020, which was followed by a sharp rebound from 2020 to 2021 (Figure 4).

### 3.5 Relationship between traffic and mortality

Yearly March and April traffic volume showed a strong positive correlation with frog individual road crossing mortality probabilities and deer–vehicle collision rates but showed only a modest correlation with salamander individual mortality probabilities (Table 4). Indeed, yearly individual frog and salamander mortality probabilities were only weakly correlated (Table 4). While many of these correlations are strong, they should be viewed with care due to their very low sample sizes (*N* = 4).

**TABLE 4:** Correlations of yearly amphibian mortality probabilities, deer collision rates, and traffic volume

	Frog mortality probability^a^	Salamander mortality probability^a^	Deer collisions^b^
Salamander mortality rate^a^	0.33		
Deer collisions^b^	0.81	0.81	
Traffic volume^c^	0.83	0.43	0.73

Abbreviation: GLMM, generalized linear mixed models.

^a^ GLMM model estimate, logit‐transformed.

^b^ ln‐transformed sum of March and April statewide collisions.

^c^ Average of ln‐transformed March and April volume/2018–2021 averages.

## 4 DISCUSSION

The influence of the COVID‐19 pandemic on ecological systems is broad and variable. Here, we demonstrate the substantial effects of reduced traffic from pandemic‐related government shutdowns on frog mortality probabilities via the MBN, a statewide community science project with four seasons of data. The dramatic 50% decline in frog mortality probabilities were strong enough to drive the overall amphibian mortality probability to be significantly lower than adjacent years. The mass breeding migrations of many amphibian populations make them far more susceptible to the hazards of road crossings than other vertebrate groups (reviewed in Trombulak & Frissell, 2000) so, conversely, the lessening of these pressures at this key time could have a greater conservation benefit. The high fatality counts that can be reached, even at limited geographic scales (Ashley & Robinson, 1996; Gibbs & Shriver, 2005), were at the very least reduced. Since roads represent significant barriers to amphibian migration and mating (Cosentino et al., 2014; Marsh et al., 2017) even small changes in pressure can have large consequences for these populations. Studies have indicated that in some species, as little as 10% annual adult mortality (Gibbs & Shriver, 2005) or just slightly fewer individuals migrating (Hels & Nachmann, 2002) may result in notable declines or extirpation within a few decades. Potentially, the reduction in traffic and community science efforts of the 2020 season will boost population sizes and buffer these populations from potential decline in the short term. Amphibian population dynamics, however, are complex and understanding the effects of a single season of reduced adult mortality may be difficult. For example, studies suggest amphibian population viability is primarily driven by juvenile survivorship, and dispersing juveniles may also be subject to heavy road mortality (Berven, 1990; Petrovan & Schmidt, 2019; Sterrett, Katz, Fields, & Campbell Grant, 2019). Given that traffic reductions were the result of a temporary shutdown, the juveniles produced from the 2020 breeding season dispersed at a time when traffic had resumed to normal levels (Osbourn, 2012; Shilling et al., 2021; Figure 4). This, coupled with naturally low survival rates (Rothermel & Semlitsch, 2006), could dampen the benefits of increased adult survival from the pandemic shutdowns. In the case of long‐lived ambystomatid salamanders (Pechmann et al., 1991), delayed sexual maturity (Semlitsch & Anderson, 2016), and incomplete annual adult participation in breeding (Madison, 1997) may mask the effects of adult mortality and not be noticeable for many years. It is certainly possible that a larger net juvenile cohort in 2020 could in turn create a larger adult breeding cohort in following years assuming no increases in other mortality sources.

It may be difficult to directly attribute amphibian population dynamics to the 2020 pandemic due to variations in maturity rates and incomplete breeding participation of adults, though a few possibilities exist. First, through continued monitoring, projects such as the MBN and others may be able to detect direct variations in migrating adults in proceeding years. Second, other projects that capture and mark juveniles may be able to evaluate the size of the 2020 juvenile cohort and how many participate in breeding as adults in subsequent years. Third, if marked 2020 juveniles are unavailable, it is possible that projects with a means to collect more intrusive data may age amphibians via examining lines of arrested growth in toe‐clipped phalanges (Sinsch, 2015) to determine the generation of captured breeding adults. We suggest special attention be paid to monitoring adult populations for the next few years to better interpret any positive effects of the COVID‐19 shutdowns on amphibians.

While our model accounts for variation in survey effort, population size fluctuations, location, and surveyor, it does not account for the time of survey. Although amphibian migrations are primarily driven by weather conditions (e.g., Brooks, Smith, Gorman, & Haas, 2019), many of our survey efforts were concentrated toward earlier night times which could result in missed migrating amphibians and subsequent mortalities later in the night. However, traffic reduces significantly throughout the night and thus mortality probabilities likely fall to near zero regardless of earlier traffic levels (Zhang et al., 2018); late night/early morning mortality probabilities were thus likely consistent in both pandemic and nonpandemic seasons since traffic would be near zero in either scenario. The time of year may also have an impact on mortality probabilities as activity levels of different species are variable throughout the year. However, our wide survey window is expected to capture all species that feature any measurable and punctual migration in the state, with explosive migrators such as the spotted salamander (*Ambystoma maculatum*), spring peeper (*Pseudacris crucifer*), wood frog, and blue‐spotted salamander providing the greatest abundance of datapoints (86% of data). Some species, such as the gray tree frog (*Hyla versicolor*) may feature migratory behavior but are much less temporally concentrated, occurring over several months later in the year (Johnson, 2005). Because of this caveat in our study design, our results should only be considered applicable to those species that migrate concurrently when traffic changes occur.

We did not anticipate the higher individual mortality probability (Table 3) and stronger link between traffic and individual mortality (Table 4) seen in frogs rather than among the slower‐moving salamanders. Mazerolle (2004) documented a much higher road mortality rate for salamanders than frogs when comparing alive versus dead amphibians. Similarly, Hels and Buchwald (2001) found that slow‐moving salamanders face the highest probability of getting killed, while fast‐moving *Lithobates* species have a lower risk. It is possible, however, that the type movement (i.e., saltation in frogs versus crawling in salamanders) rather than speed affects the probability of physical impact with a vehicle; the effective surface area of a vehicle (e.g., undercarriage, bumpers, tires) is likely greater for a jumping animal than a crawling one, however this seems unlikely as these effective areas are likely not so drastically different between the vehicles in our study area and the study areas of the aforementioned references. We also considered the potential for observation bias by volunteers. For example, living frogs are cryptically colored dorsally whereas the white venter of dead frogs is more conspicuous, potentially leading to under‐representation of live frogs in surveys and thus creating higher frog mortality estimates. Coloration of local salamanders are essentially the opposite, with most having bold coloration on the dorsum and cryptic colors on the venter, potentially biasing toward detecting live individuals and thus lower mortality estimates should roadkill salamanders land with anything but the dorsum exposed. Our data, however, do not support this claim; we did not observe a difference between cryptically colored (e.g., spring peepers, four‐toed salamanders [*Hemidactylium scutatum*]) and brightly colored (e.g., spotted salamanders, green frogs) amphibians.

Our other finding that frog mortality, but not salamander, was positively associated with precipitation suggests these environmental factors may influence frog versus salamander movements differently. Sexton, Phillips, and Bramble (1990) observed that salamander movements do not have a strong positive relationship with precipitation; rather, nearly all movements occurred after a relatively low threshold of precipitation (0.4 cm) and did not increase with higher precipitation amounts. Conversely, frogs may not reach this threshold as quickly and activity may increase substantially throughout the migration season. For example, activity levels of the spring peeper, the most encountered amphibian in this study, increase consistently with precipitation (Kirlin, Gooch, Price, & Dorcas, 2006) and temperature (Taigen, O'Brien, & Wells, 1996), as is also the case with wood frogs (Bellis, 1962; Heatwole, 1961) and the common toad (*Bufo bufo*) (Arnfield, Grant, Monk, & Uller, 2012). If salamanders reach this threshold well before frogs, this could explain our observed difference as activity levels continue to increase for frogs (e.g., more likely to be active or in roads) but no longer increase for salamanders. If salamanders reach the activity‐environment threshold quickly, as Sexton et al. (1990) suggest, we will need drier days to see an effect in salamanders. Interestingly, temperature did not show strong influence on mortality probabilities despite temperature being well‐known as a major influence of activity in amphibians (e.g., Taigen et al., 1996). This may be because the measure we used, maximum daily temperature, may not be well correlated with the actual temperature during migrations and thus a more temporally accurate metric may be necessary.

We supported our principal study on the effects of pandemic‐related declines in amphibian road mortality by examining other wildlife collision data gathered by the MDOT given the relatively short time span of our dataset. We again saw half as many vehicle strikes for white‐tailed deer in 2020 versus 2019 (Figure 3). Although relatively fewer collisions were recorded, the pattern of 50% or greater reduction held for all wildlife recorded, including wild turkey and moose. Other reports of reduced road mortality during the pandemic with similar effects to ours have been noted in other species. For example, a 58% decline in mountain lion road mortality in California (Shilling et al., 2021), a 48% reduction in marsupial road mortalities in Tasmania (Driessen, 2021), a 50% decline in amphibian road mortality in Italy (Manenti et al., 2020), and an approximately 50% decline in hedgehog mortality in Poland (Lopucki, Kitowski, Perlinska‐Teresiak, & Klich, 2021).

Findings from our study can be considered a “stress test” (Bates et al., 2021) that demonstrate clear, tangible changes from altered human behavior recorded by a growing cohort of volunteers. With such clear changes, easily identifiable actions to achieve reduced mortality, and a growing participation base, it is possible this project and others like it may inspire wider reaching social changes such as driving more mindfully (e.g., reducing trips and limiting travel during amphibian breeding), or lobbying for wildlife crossing corridors or ecologically sensitive construction projects. It is well known that roads and associated traffic are major sources of mortality for amphibians and other wildlife. Our study shows that while high road mortality can be reduced significantly by changes in human behavior, these changes will likely be difficult to replicate in the near future without another major social catalyst. The authors would also like to emphasize that while the lockdowns have provided a great opportunity to examine phenomena such as this, one should approach and share their ideas and findings with sensitivity and respect as the loss of human life is large and continues to grow as the pandemic is still occurring worldwide at the time of writing.

## CONFLICT OF INTEREST

The authors declare no conflict of interest.

## AUTHOR CONTRIBUTIONS

**Gregory LeClair**: Provided the primary data, primary writing/editing responsibilities, literature review, and coordination and is the founder and coordinator of MBN. **Matthew W. H. Chatfield**: Gathered and analyzed environmental data and provided literature review, edits, and writing and serves as a scientific advisor for MBN. **Zachary Wood**: Provided statistical analysis, figures, statistical methods writing, and edits. **Jeffrey Parmelee**: Communicated with wildlife agencies, gathered traffic data, and provided writing and edits and serves as a scientific advisor for MBN. **Cheryl A. Frederick**: Provided overarching edits and conceptual guidance and serves as a scientific advisor for MBN.

## ETHICS STATEMENT

This community science project did not include animal experimentation or invasive manipulation of any form. Interactions with animal subjects were brief and typically involved counting from a distance and occasionally directly transporting live individuals across roads in the direction of travel with gloved or sterile hands. Volunteers were trained by field professionals in proper handling techniques to ensure fast and safe transport.

## Supporting information


**Appendix S1**: Supporting information

**Appendix S2**: Supporting information

**Appendix S3**: Supporting information

**Appendix S4**: Supporting information

**Appendix S5**: Supporting information

**Appendix S6**: Supporting information

## Data Availability

Data and relevant files are available as Supporting Information files.
