# Supplementary material for: Influence of the COVID‐19 pandemic on amphibian road mortality
Source: Conserv Sci Pract. 2021 Sep 29;3(11):e535. doi: 10.1111/csp2.535 (PMC8646393; doi:10.1111/csp2.535)
Supplement: Supplementary file 1 — Appendix S1: Supporting information [file CSP2-3-e535-s003.pdf]

Appendix One: Species-Specific Mortality Probabilities

We examined species-specific individual mortality probabilities of all species for which we found at least 50 individuals (alive or dead) in total across all four study years. We fit the following generalized linear binomial mixed model using the *lme4* package in R version 4.1.0 (Bates et al. 2015; R Core Team 2021):
(A1.1)

$$M = \beta_S + \beta_Y + \beta_{SC}C + \beta_{SP}P + \sigma^2_{Site} + \sigma^2_{Recorder}$$

M is individual mortality probability; β_S is a species-specific (S) mortality probability (fixed effect); β_Y is a year-specific (Y) mortality probability (fixed effect); $\beta_{SC}C$ is a species-specific fixed effect of maximum temperature (C); $\beta_{SP}P$ is a species-specific fixed effect of daily precipitation (P); and σ^2_{Site} and $\sigma^2_{Recorder}$ are random effects for site and recorder, respectively. C and P were scaled (set to mean = 0 and sd = 1) for this analysis.

We used Type II likelihood ratio tests to test for significant effects of species, species-specific effects of temperature, and species-specific effects of precipitation on individual mortality probability.

We found significant species-specific individual mortality probabilities, as well as significant species-specific effects of temperature on individual mortality probabilities and borderline significant species-specific effects of precipitation on individual mortality probabilities (Table A1.1).

Table A1.1. Type II likelihood ratio tests for species-specific individual mortality probabilities and effects of temperature and precipitation.

**Variable**	χ^2	**df**	***p***
Species	137.27	11	< 0.001
Species-specific effect of temperature	30.81	11	0.001
Species-specific effect of precipitation	18.60	11	0.069

Bullfrogs, blue-spotted salamanders, red-backed salamanders, four-toed salamanders, gray tree frogs, and spotted salamanders had lower individual mortality probabilities than other species (Figure A1.1). Species-specific effects of temperature and precipitation on individual mortality probabilities were highly variable (Figure A1.1).

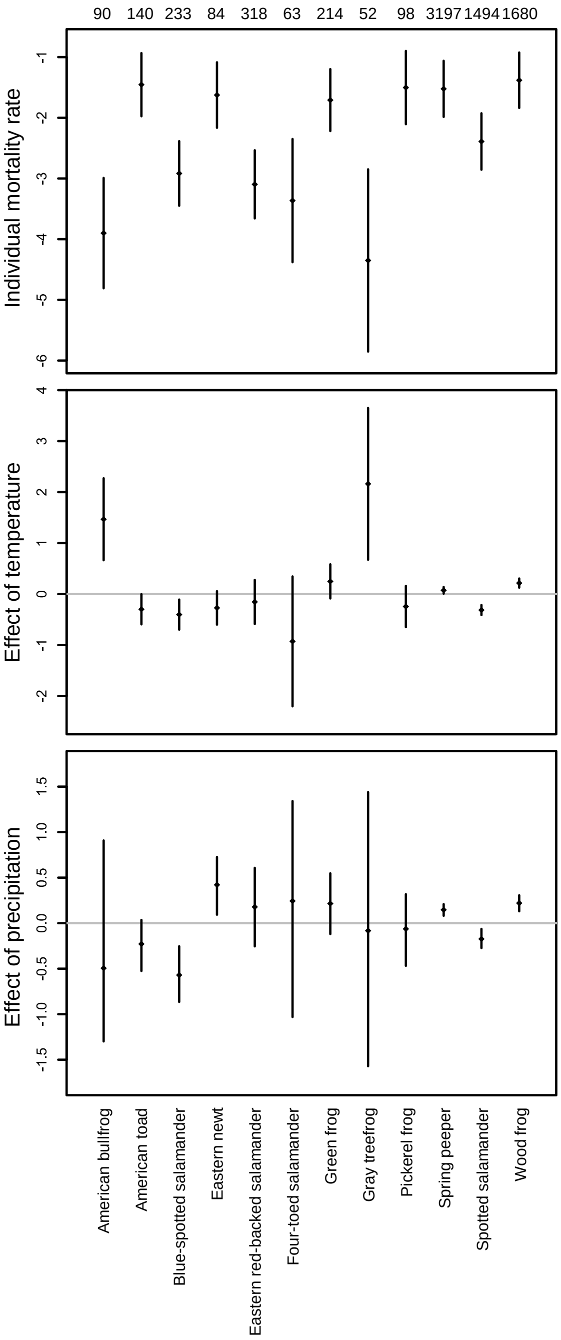

Figure A1.1. Species-specific individual road crossing mortality probabilities and effects of temperature and precipitation on individual mortality probabilities. Points and lines show binomial generalized linear mixed model estimates and standard errors, respectively, in logit-transformed space. Estimates are for average probabilities across all four study years. Numbers above the figure indicate sample sizes for each species.

References

Bates D, Mächler M, Bolker B, Walker S (2015). Fitting linear mixed-effects models using lme4. Journal of Statistical Software, *67*(1), 1–48.

R Core Team (2021). R: A language and environment for statistical computing. R Foundation for Statistical Computing, Vienna, Austria.
