# Supplementary material for: Influence of the COVID‐19 pandemic on amphibian road mortality
Source: Conserv Sci Pract. 2021 Sep 29;3(11):e535. doi: 10.1111/csp2.535 (PMC8646393; doi:10.1111/csp2.535)
Supplement: Supplementary file 6 — Appendix S6: Supporting information [file CSP2-3-e535-s006.pdf]

Appendices

Appendix One: Species-Specific Mortality Probabilities

We examined species-specific individual mortality probabilities of all species for which we found at least 50 individuals (alive or dead) in total across all four study years. We fit the following generalized linear binomial mixed model using the *lme4* package in R version 4.1.0 (Bates et al. 2015; R Core Team 2021):
(A1.1)

$$M = \beta_S + \beta_Y + \beta_{SC}C + \beta_{SP}P + \sigma^2_{Site} + \sigma^2_{Recorder}$$

M is individual mortality probability; β_S is a species-specific (S) mortality probability (fixed effect); β_Y is a year-specific (Y) mortality probability (fixed effect); $\beta_{SC}C$ is a species-specific fixed effect of maximum temperature (C); $\beta_{SP}P$ is a species-specific fixed effect of daily precipitation (P); and σ^2_{Site} and $\sigma^2_{Recorder}$ are random effects for site and recorder, respectively. C and P were scaled (set to mean = 0 and sd = 1) for this analysis.

We used Type II likelihood ratio tests to test for significant effects of species, species-specific effects of temperature, and species-specific effects of precipitation on individual mortality probability.

We found significant species-specific individual mortality probabilities, as well as significant species-specific effects of temperature on individual mortality probabilities and borderline significant species-specific effects of precipitation on individual mortality probabilities (Table A1.1).

Table A1.1. Type II likelihood ratio tests for species-specific individual mortality probabilities and effects of temperature and precipitation.

Variable	χ^2	df	p
Species	137.27	11	< 0.001
Species-specific effect of temperature	30.81	11	0.001
Species-specific effect of precipitation	18.60	11	0.069

Bullfrogs, blue-spotted salamanders, red-backed salamanders, four-toed salamanders, gray tree frogs, and spotted salamanders had lower individual mortality probabilities than other species (Figure A1.1). Species-specific effects of temperature and precipitation on individual mortality probabilities were highly variable (Figure A1.1).

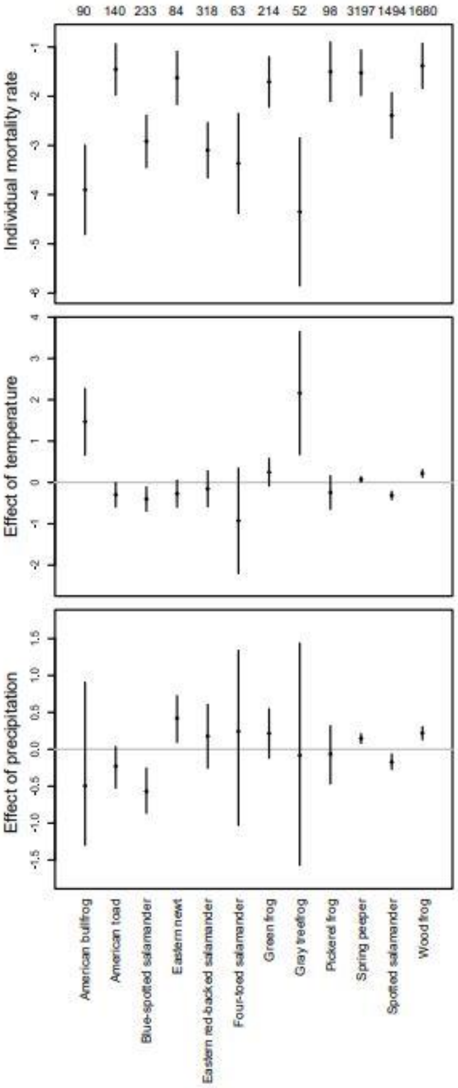

Figure A1.1. Species-specific individual road crossing mortality probabilities and effects of temperature and precipitation on individual mortality probabilities. Points and lines show binomial generalized linear mixed model estimates and standard errors, respectively, in logit-transformed space. Estimates are for average probabilities across all four study years. Numbers above the figure indicate sample sizes for each species.

References
Bates D, Mächler M, Bolker B, Walker S (2015). Fitting linear mixed-effects models using lme4. Journal of Statistical Software, *67*(1), 1–48.

R Core Team (2021). R: A language and environment for statistical computing. R Foundation for Statistical Computing, Vienna, Austria.

Appendix Two: Number of amphibians and amphibian mortality recorded by Maine Big Night volunteers from 2018–2021.

Species	2018	2019	2020	2021	Total	Mortality Rate

Anura						
Anaxyrus americanus	0	1	34	106	141	0.1986
Hyla versicolor	0	0	1	51	52	0.1731
Lithobates catesbeianus	0	0	2	88	90	0.0556
L. clamitans	1	1	67	145	214	0.2477
L. palustris	0	0	1	97	98	0.3163
L. pipiens	0	0	20	5	25	0.3200
L. septentrionalis	0	0	0	2	2	0.0000
L. sylvaticus	18	59	345	1271	1693	0.2688
Pseudacris crucifer	8	170	611	2417	3206	0.2955
Caudata						
Ambystoma laterale	17	31	53	134	235	0.0894
A. maculatum	5	43	439	1012	1499	0.1388
Desmognathus fuscus	0	0	0	5	5	0.000
Eurycea bislineata	0	0	0	5	5	0.000
Hemidactylium scutatum	0	0	13	50	63	0.0318
Notophthalmus viridescens	0	6	24	56	86	0.2976
Plethodon cinereus	0	2	37	297	336	0.0387
Total Amphibians	49	313	1646	5741	7749	

Appendix S3. Wildlife collisions as reported by the Maine Department of Transportation in April from 2010–2021.

Species	2010	2011	2012	2013	2014	2015	2016	2017	2018	2019	2020	2021	Total
Odocoileus virginianus	120	207	128	245	334	458	229	419	494	441	234	287	5016
Alces alces	39	13	19	9	9	10	7	10	10	10	5	10	313
Meleagris gallopavo	7	6	8	12	11	14	11	31	18	26	11	17	227
Ursus americanus	2	0	1	1	0	1	0	2	2	1	1	1	14
Other Animals	6	11	9	3	11	7	8	11	13	14	6	9	157
Total Animals	174	237	165	270	365	490	255	473	537	492	257	yee	5403
